# Tactile Feedback in Robot‐Assisted Minimally Invasive Surgery: A Systematic Review

**DOI:** 10.1002/rcs.70019

**Published:** 2024-12-07

**Authors:** Jacinto Colan, Ana Davila, Yasuhisa Hasegawa

**Affiliations:** ^1^ Department of Micro‐Nano Mechanical Science and Engineering Nagoya University Nagoya Japan; ^2^ Institutes of Innovation for Future Society Nagoya University Nagoya Japan

**Keywords:** haptic interface, minimally invasive surgery, robotic assisted surgery, tactile feedback

## Abstract

**Background:**

Robot‐assisted systems have predominantly relied on teleoperation, where visual feedback is the primary source of information. However, advances in tactile sensing and displays offer new opportunities to enhance surgical transparency, efficiency, and safety.

**Methods:**

A PRISMA‐guided search was conducted across PubMed, IEEE Xplore, Scopus, and Web of Science databases to identify relevant studies.

**Results:**

Out of 645 screened articles, 98 met the inclusion criteria, and 33 were included in the final review. The review discusses various tactile feedback stimulus types, applications, and challenges in the context of robot‐assisted minimally invasive surgery.

**Conclusion:**

While kinaesthetic feedback has been extensively explored to restore the natural interaction between the surgeon and the surgical environment, tactile feedback remains largely confined to research settings. This is due to significant challenges in integrating tactile feedback into robotic systems and current limitations of sensing technologies.

## 1 Introduction

Recent advancements in robot‐assisted surgery technologies have significantly addressed the limitations of traditional minimally invasive surgery (MIS). Robot‐assisted minimally invasive surgical (RMIS) systems have enhanced surgeon capabilities in various surgical areas by offering improved depth perception [1], intuitive human‐robot interfaces [2], highly dexterous surgical instruments [3], and Remote Center of Motion (RCM) mechanisms that minimise tissue trauma and improve precision of surgical manoeuvres [4, 5]. These technological advances collectively extend the benefits of traditional MIS by reducing both the cognitive load and the physical strain on surgeons.

Despite significant developments, RMIS still faces a crucial limitation: the lack of haptic sensation, including both force and tactile feedback. In open surgery, haptic feedback is essential, allowing surgeons to differentiate between healthy and pathological tissues, manipulate delicate structures, and identify organs by touch. The lack of this sensory input in RMIS can negatively impact surgical performance and patient outcomes, with studies indicating a strong correlation between the absence of haptic feedback and an increased risk of surgical complications [6]. This limitation is particularly concerning in delicate procedures, where precise force application and tissue discrimination are essential to avoid intraoperative injuries.

To address this gap, there has been growing interest in the development of force and tactile sensors designed for surgical applications. These sensors aim to capture subtle tactile and force information that is naturally available to surgeon's hands during open procedures. However, comparatively less attention has been directed towards developing effective feedback display systems that can convey this sensory information to surgeons in a meaningful and intuitive manner during robotic procedures.

This systematic review aims to synthesise recent progress in the development of tactile feedback technologies specifically designed for minimally invasive surgical applications. By examining the current state of the art in tactile feedback systems, this paper seeks to:

- Identify innovative approaches to providing tactile feedback in RMIS.
- Assess the effectiveness of various tactile feedback modalities across different surgical applications.
- Discuss the specific challenges encountered in the development and implementation of these technologies.

### 1.1 Tactile Feedback in Minimally Invasive Surgery

Haptic feedback systems in minimally invasive surgery (MIS) are generally classified into two primary types: kinaesthetic (or force) feedback and tactile (or cutaneous) feedback [7]. The distinction between these two types lies in the nature of the sensory information they convey [8]. Kinaesthetic feedback provides information related to position, velocity, and force exerted on objects, relying on mechanoreceptors located within joints and muscles. In traditional MIS, some degree of kinaesthetic feedback is retained through the transmission of forces along the shaft of surgical instruments. However, in robot‐assisted minimally invasive surgery (RMIS), this feedback is significantly reduced or completely absent. Surgeons in RMIS must rely predominantly on visual cues, such as tissue deformation, to estimate interaction forces [9, 10].

In contrast, tactile feedback involves the perception of surface properties, including texture, temperature, and pressure distribution, commonly referred to as the sense of touch. This feedback is generated by stimulation of mechanoreceptors located on the skin, which respond to various stimuli such as pressure, stretching, and vibrations. Unlike kinaesthetic feedback, tactile feedback is completely lost in both MIS and RMIS. This loss is particularly pronounced in RMIS, where the surgeon is physically decoupled from the patient, and no additional channel is available to transmit tactile information to the surgeon.

**FIGURE 1:**
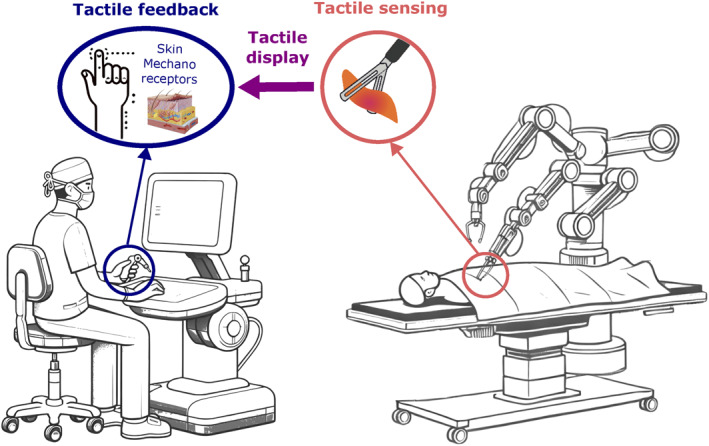
Tactile feedback in robot‐assisted minimally invasive surgery is achieved by converting tactile stimuli into data via tactile sensors. This data is sent to a tactile display embedded in the human‐robot interface, recreating the feedback sensation on the human skin mechanoreceptors. Relevant works can mainly be categorised into two groups depending on the feedback modality: Skin deformation and Vibrotactile devices.

The challenge of recreating tactile feedback in RMIS is compounded by the complexity and variety of tactile sensations perceived by the human hand. Displaying this information in a meaningful way remains a significant hurdle. Figure 1 illustrates the workflow of a tactile feedback system designed for minimally invasive surgery. In this process, tactile stimuli are converted into data via tactile sensors, which are then transmitted to a tactile display embedded in the human‐robot interface. This display recreates the tactile sensation on the mechanoreceptors of the surgeon's skin, simulating the experience of touch.

The distinction between kinaesthetic and tactile feedback devices is not always well defined, as many devices integrate both types of feedback, especially in systems where tactile feedback is embedded within the user interface. In this review, we focus on devices that prioritise tactile feedback, omitting those that provide only kinaesthetic feedback or single non‐distributed force feedback, as these have been extensively covered in previous literature.

### 1.2 Tactile Sensing

Research on force and tactile sensing has been extensively reviewed in the literature, with numerous studies highlighting new technologies designed to overcome the specific challenges posed by the MIS workspace [11, 12, 13, 14, 15]. The development of tactile sensing systems involves three main components: transduction of tactile data, processing of these data to extract useful information, and display of this information to the surgeon. Various transducer technologies, such as those based on displacement, current, pressure, resistance, capacitance, piezoelectric effects, vibration, and optics, have been explored for their potential to provide both normal and shear force sensitivity [12]. Reliable and robust tactile sensing is a fundamental requirement for the seamless generation of tactile feedback in MIS applications.

### 1.3 Related Surveys

Several surveys have reviewed the current state of haptic technologies [16, 17, 18, 19]. However, the focus has predominantly been on kinaesthetic feedback, with only partial attention given to tactile feedback. In addition, these reviews often overlook the specific requirements of RMIS, such as integration into human‐robot interfaces. Schostek, Schurr and Buess [8] provided a comprehensive review of tactile feedback for laparoscopic surgery. However, significant developments in tactile sensing and feedback technologies have occurred since its publication, necessitating an updated examination. More recent reviews have addressed tactile displays for conventional laparoscopic surgery [20]. However, RMIS presents additional challenges due to its reliance on teleoperation interfaces. Okamura [21] reviewed haptic devices for general applications without specifically discussing the unique requirements and constraints of surgical applications. Similarly, Pacchierotti et al. [22] have provided a comprehensive overview of tactile feedback for general‐purpose teleoperated robotic systems without specifically targeting RMIS applications.

This systematic review aims to address the gaps in the literature by presenting an updated overview of the advances in tactile feedback devices for RMIS over the last decade. Unlike previous works, this review will explicitly address the challenges and requirements unique to tactile feedback in RMIS, considering the complexities introduced by human‐robot interfaces in teleoperated surgical systems.

## 2 Materials and Methods

### 2.1 Research Question

The research question for this systematic review is defined using the Population, Interventions, Comparators, and Outcomes (PICO) framework [23], as shown in Table 1. The PICO framework ensures a comprehensive and systematic approach to identifying relevant studies, guiding the selection criteria for inclusion and exclusion.

**TABLE 1:** PICO framework for defining the research question and inclusion/exclusion criteria.

	Inclusion criteria	Exclusion criteria
Population	Studies focused on robot‐assisted minimally invasive surgery (RMIS)	Studies focusing on other types of MIS, such as conventional laparoscopic surgery
Intervention	All forms of tactile feedback technologies (e.g., skin deformation, vibrotactile devices)	Haptic devices that provide other forms of feedback only (e.g., kinaesthetic feedback without tactile feedback)
Comparators	Not applicable	—
Outcomes	Studies that render tactile information from MIS tasks in ex vivo, phantom, or virtual setups	Studies that do not include MIS‐related tasks or do not experimentally validate tactile feedback

The research question identified for this review is as follows: *In robot‐assisted minimally invasive surgery (RMIS), which tactile feedback technologies have been proposed and experimentally validated for the restoration of tactile sensations during RMIS tasks?*

By focusing on technologies that have been experimentally validated in RMIS tasks, this review aims to provide a comprehensive understanding of the current state of tactile feedback systems and the challenges associated with their integration into RMIS platforms.

### 2.2 Search Methodology and Systematic Review

**FIGURE 2:**
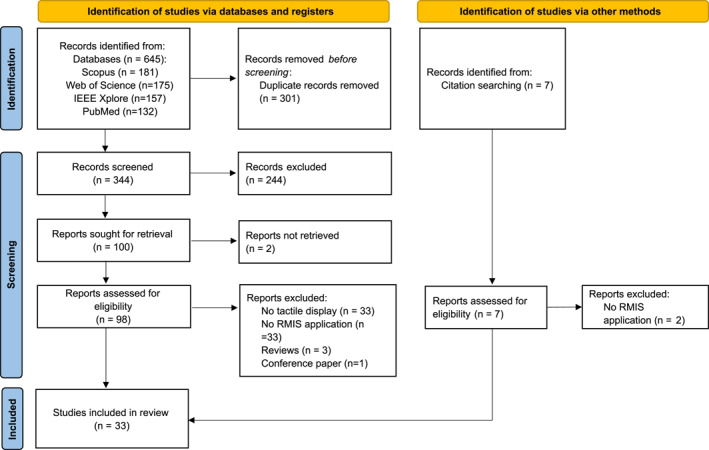
Flow diagram showing the study selection process for this systematic review.

This systematic review was conducted in accordance with the Preferred Reporting Items for Systematic Reviews and Meta‐Analyses (PRISMA) guidelines [24], ensuring a transparent and reproducible methodology. The study selection process is illustrated in Figure 2. We performed a comprehensive literature search across four major databases: Web of Science, Scopus, IEEE Xplore Digital Library, and PubMed. The search covered the period from January 2014 to December 2024 to capture the most recent developments in tactile feedback technologies for robot‐assisted minimally invasive surgery (RMIS). The search strategy used a combination of keywords related to RMIS and tactile feedback. The search terms are detailed in Table 2.

**TABLE 2:** Search strategy.

Search strategy
TITLE‐ABS‐KEY ( ( (robot* AND ((”minimally invasive”) OR laparoscop* OR endoscop* OR mis OR surg*) ) OR rmis OR ramis) AND (somatosensory OR tactile OR vibrotactile OR cutaneous) AND (display OR feedback OR stimul* OR augmentation OR interface OR device) ) AND PUBYEAR > 2013

This study involved the analysis of publicly available data and did not involve human participants or animals, so ethical approval was not required.

### 2.3 Study Selection and Inclusion Criteria

In this study, we considered only articles published in English, excluding conference proceedings and those with duplicate titles at the initial stage of the selection process. During the screening phase, titles and abstracts were carefully reviewed to eliminate non‐peer‐reviewed publications, such as short essays, general discussions, posters, and project or workshop proposals. We also excluded articles not related to tactile feedback technologies in robot‐assisted minimally invasive surgery (RMIS), as well as surveys and review articles. Articles without full‐text availability were also removed from consideration. Our focus was specifically on studies investigating tactile feedback in RMIS, and we included only those that provided experimental validation, whether in virtual environments, phantom models, or ex vivo experiments. Studies related to conventional MIS or those not involving tactile feedback technologies were excluded.

Our database search across Scopus, Web of Science, IEEE Xplore, and PubMed yielded a total of 645 articles (Scopus: 181, Web of Science: 175, IEEE Xplore: 157, PubMed: 132). After removing 301 duplicates, 344 articles remained for screening. Upon reviewing the titles and abstracts, 244 articles were excluded for various reasons: 3 were not peer‐reviewed, 217 were irrelevant to the topic, and 24 were surveys or reviews. Additionally, 2 articles could not be retrieved. This left 98 articles eligible for full‐text review. Of these, 33 did not include tactile feedback technology, 33 lacked experimental validation in RMIS, and 4 were further excluded as they were review articles or conference proceedings. Furthermore, 7 records were identified through citation searching, with 2 being excluded for not involving RMIS applications. Ultimately, 33 articles met all criteria and were selected for inclusion in this review.

## 3 Results

### 3.1 Descriptive Analysis if the Reviewed Studies

**FIGURE 3:**
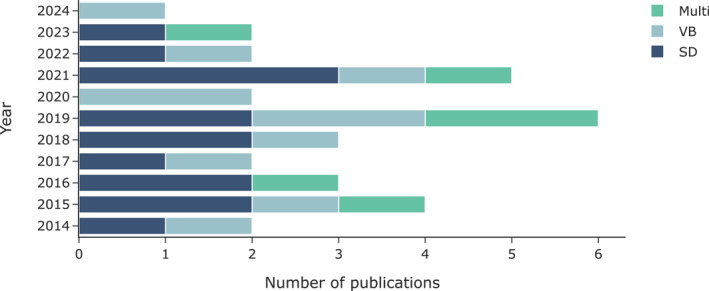
Number of publications per year over the last 10 years, categorised according to the tactile feedback modality employed. Multi, Multimodal; SD, Skin deformation; VB, Vibrotactile.

**FIGURE 4:**
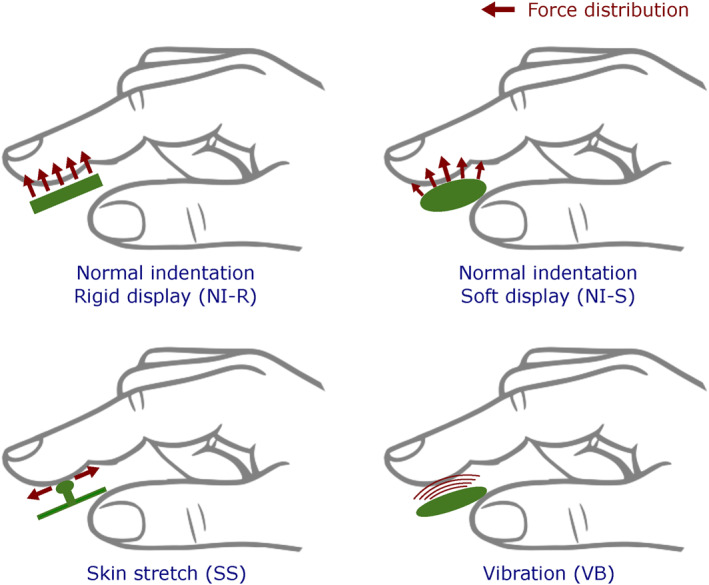
Schematic of the multiple tactile stimulus modalities studied in the literature.

Figure 3 illustrates the distribution of publications over the past decade, categorised by the tactile feedback modality employed in the studies. Tactile feedback modalities can be broadly categorised based on the technical approach used in the display: skin deformation (SD), which includes normal indentation with rigid (NI‐R) and soft indenters (NI‐S), as well as skin stretch (SS); vibrotactile (VB); and multimodal approaches that combine skin deformation and vibrotactile feedback (Multi). A schematic representation of these tactile stimulus modalities is provided in Figure 4.

The data suggest a consistent interest in tactile feedback technologies for RMIS over the past decade. Among the various modalities, skin deformation (SD) devices have received significant attention, as evidenced by the consistent number of publications in this category each year. This indicates that SD has been a primary focus for researchers, likely due to their ability to provide a more intuitive and predictable tactile experience, as the deformation of the skin directly correlates with the force or pressure applied.

The following sections will explore relevant work within these categories, highlighting the technological innovations and experimental validations in RMIS.

### 3.2 Skin Deformation Based Tactile Feedback

Skin deformation has been widely explored as a mechanism for sensory substitution in RMIS, offering the ability to convey both magnitude and directional information related to environmental properties such as stiffness and friction. Tactile displays that utilise skin deformation can be classified into three categories based on the type of indentor used: normal indentation with rigid displays, normal indentation with soft displays, and lateral skin stretch.

#### 3.2.1 Normal Indentation With Rigid Displays (NI‐R)

The use of normal indentation with rigid displays leverages the compliant nature of the fingertip. When a rigid platform displaces against the fingertip, it creates a contact stress distribution that simulates the sensation of touching or pressing against different materials. An overview of NI‐R related studies is presented in Table 3.

**TABLE 3:** Summary of studies utilising rigid tactors for skin deformation‐based (NI‐R) tactile feedback in RMIS.

Reference	Tactile stimulus	Actuator	Additional stimulus	Location	Robotic interface	Robotic system	Application	Sensor	Virtual	Phantom	Ex vivo
Meli, Pacchierotti and Prattichizzo [25]	NI‐R	DC motor	K, V, A	Finger	Omega 7 (Force Dimension, Switzerland)	Virtual	TM	None	✓	—	—
Pacchierotti et al. [26]	NI‐R	DC motor	K	Finger	Omega 3 (Force Dimension, Switzerland)	Customised robot (KR3, KUKA, Germany)	NI	Force sensor (FSR 400, Interlink Electronics, USA)	—	✓	—
Chinello et al. [27]	NI‐R	Servomotor	K, V	Finger	Falcon (Novint, USA)	Customised robot (UR5, Universal Robot, Denmark)	PP	F/T sensor (Nano25, ATI, USA)	✓	✓	—
Cabibihan et al. [28]	NI‐R	DC motor	V	Finger	Geomagic Touch (3D Systems, USA)	1‐DoF durgical grasper	PP	Force sensor (FlexiForce B201, Tekscan, USA)	—	✓	—

Abbreviations: A, auditory; K, kinaesthetic; NI, needle insertion; NI‐R, normal Indentation (rigid indenter); PP, palpation; TM, telemanipulation; V, visual.

**FIGURE 5:**
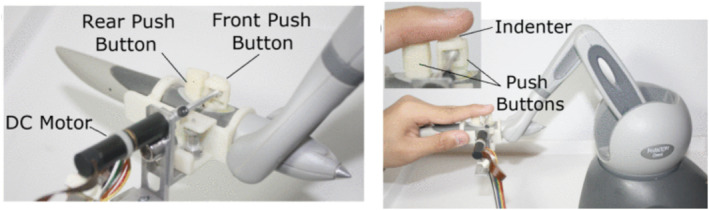
Tactile feedback with normal indentation (NI‐R). Left: Force feedback device components featuring a rigid indenter. Right: Subject's finger interacting with the device indenter. The inset shows the indenter‐fingertip contact. Adapted from Cabibihan et al. [28].

Meli, Pacchierotti and Prattichizzo [25] investigated the use of fingertip skin deformation devices in a teleoperated surgical setup. The devices, driven by small electrical motors, move a platform towards the user's fingertip to provide normal indentation feedback during a 7‐DoF bimanual peg board task. This approach significantly improved task performance metrics, including completion time, grip forces, and ring displacement, even under a 20 ms communication delay. Building on this concept, Pacchierotti et al. [26] introduced a method to enhance passive teleoperation systems by integrating kinaesthetic and cutaneous haptic feedback through a wearable 3‐DoF cutaneous device. This device applies forces directly to the fingertip by controlling cable lengths and tilting a mobile platform. During teleoperated needle insertion in a soft tissue phantom, this combined feedback approach led to significant improvements in perceived stiffness and overall task performance. A similar approach is proposed in [27], with a modular wearable finger interface, which combines a 3‐DoF fingertip cutaneous device with a 1‐DoF finger kinaesthetic exoskeleton. This interface uses servo motors to actuate a mobile platform that can press into and re‐angle against the fingertip, providing both normal and shear forces. In a robot‐assisted palpation task, this combined cutaneous and kinaesthetic feedback significantly improved the detection of a hidden stiff sphere within a simulated tissue model, outperforming setups with no feedback or cutaneous feedback alone. In addition to these studies, research has also focused on the influence of multimodal feedback on surgical tasks [28]. A sensorized surgical grasper combined with a fingertip NI‐R device was used to evaluate the effects of visual and haptic feedback on detecting threshold forces during a surgical grasping task (Figure 5). The results showed that the integration of visual and haptic cues significantly improved precision by reducing threshold force detection, demonstrating the effectiveness of combining multiple sensory modalities in minimally invasive robotic surgery.

Despite their effectiveness in conveying force information, normal indentation with rigid displays faces several important limitations. Rigid displays usually involve keeping platforms in contact with the surface of the skin which can lead to discomfort, skin irritation, and even pain, particularly during prolonged use or when applying high forces [25]. Furthermore, the rigidity of these displays can also limit their ability to conform to the natural curvature of the skin, resulting in a reduced contact area and decreased sensitivity [28]. Additionally, the mechanical properties of rigid displays can also introduce unwanted vibrations, noise, and mechanical artefacts, which can further degrade the tactile experience.

#### 3.2.2 Normal Indentation With Soft Displays (NI‐S)

**FIGURE 6:**
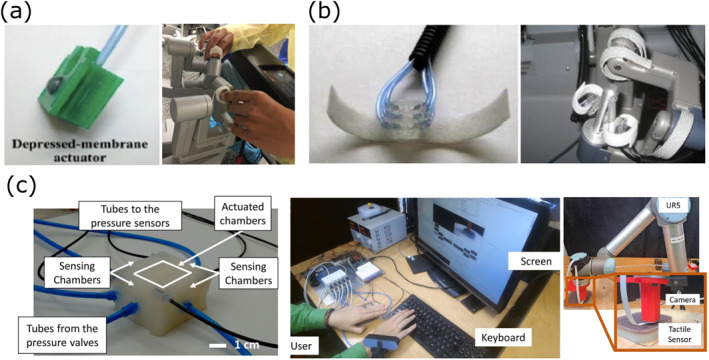
Tactile feedback with normal indentation (NI‐S). (a) Depressed‐membrane actuator integrated into the da Vinci surgeon console. Adapted from Juo et al. [9] (ⓒ 2022 Springer Nature, reprinted with permission). (b) Pneumatic actuator for tactile feedback on the da Vinci robot. Adapted from Wottawa et al. [29] (ⓒ 2016 Springer Nature, reprinted with permission). (c) Soft physical twin for remote palpation tactile feedback. Adapted from Costi and Iida [33].

Soft displays, particularly those using pneumatic actuators, have been explored as a means to provide tactile feedback in RMIS by simulating the sensation of tissue properties such as stiffness and force. These systems use soft materials to create a more natural and nuanced tactile experience, which can be critical for tasks requiring delicate manipulation and precise force control. A summary of previous research on NI‐S devices is presented in Table 4, and illustrative examples are depicted in Figure 6.

**TABLE 4:** Summary of studies utilising soft displays for skin deformation‐based tactile feedback in RMIS.

Reference	Tactile stimulus	Actuator	Additional stimulus	Location	Robotic interface	Robotic system	Application	Sensor	Virtual	Phantom	Ex vivo
Wottawa et al. [29]	NI‐S	Pneumatic actuator	V	Finger	da Vinci console interface	da Vinci robot (Intuitive Surgical, USA)	TM	Piezoresistive force sensors	—	✓	✓
Abiri et al. [30]	NI‐S	Pneumatic actuator	V	Finger	da Vinci console interface	da Vinci robot (Intuitive Surgical, USA)	TM	Piezoresistive force sensors	—	✓	—
Li, Konstantinova and Althoefer [31]	NI‐S	Pneumatic actuator	K, V	Finger	Phantom Omni (3D Systems, USA)	Customised robot (M‐6iB, FANUC, Japan)	PP	F/T sensor (Nano17, ATI, USA)	—	✓	—
Juo et al. [9]	NI‐S	Pneumatic actuator	V	Finger	da Vinci console interface	da Vinci robot (Intuitive Surgical, USA)	TM	Force sensor (FlexiForce B201, Tekscan, USA)	—	—	✓
Doria et al. [32]	NI‐S	DC motor	—	Finger	Geomagic Touch (3D Systems, USA)	In‐house 2‐DoF indenting system	PP	F/T sensor (Nano17, ATI, USA)	—	✓	✓
Costi and Iida [33]	NI‐S	Pneumatic actuator	V	Finger	Keyboard, Physical twin	Customised robot (UR5, Universal Robot, Denmarks)	PP	3DoF force sensor (uSkin 4 × 4, Xela Robotics, Japan)	—	✓	—

Abbreviations: K, kinaesthetic; NI‐S, normal indentation (soft display); PP, palpation; TM, telemanipulation; V, visual.

Wottawa et al. [29] introduced a pneumatic display system designed to stimulate the surgeon's fingertips by adjusting air pressure in hemispheric silicone balloons (Figure 6b). The system was integrated with piezoresistive sensors mounted at the tip of a da Vinci robot forceps, allowing the grasping forces to be transmitted to the surgeon's fingertips. Their experiments, conducted in an ex vivo porcine bowel model, demonstrated that this tactile feedback system significantly reduced the applied grasping forces, thereby minimising tissue damage during robotic manipulation. Similarly, Abiri et al. [30] utilised piezoresistive force sensors installed on the graspers of the da Vinci IS 1200 system to provide tactile feedback through pneumatic actuators that applied normal forces to the surgeon's fingertips. This system was evaluated during a peg transfer task involving 45 novice subjects. The study revealed that the introduction of tactile feedback effectively mitigated the impact of visual‐perceptual mismatches on task performance, leading to fewer peg drops and shorter completion times. Li et al. [31] proposed a haptic feedback system using pneumatic actuators to convey soft tissue stiffness information during RMIS. Their system featured a deformable surface with a soft silicone layer and a silicone rubber membrane, which provided a more realistic representation of tissue stiffness. Experimental results showed that this system improved sensitivity and reduced detection time for identifying hard nodules, outperforming approaches that relied solely on pseudo‐haptic feedback. More recently, Juo et al. [9] developed a pneumatic tactile feedback system to provide graded tactile feedback to the surgeon's fingertips during robotically assisted total mesorectal excision in a porcine model (Figure 6a). Their in vivo study found that this tactile feedback system significantly reduced retraction forces, suggesting the potential to decrease tissue damage during robotic surgery. However, the system did not significantly affect dissection forces, indicating that while tactile feedback is beneficial, its effects may vary depending on the specific surgical task. Costi and Iida [33] explored the concept of a soft robotic bilateral physical twin for remote palpation, employing pneumatic chambers to provide distributed tactile feedback (Figure 6c). Their comparative study demonstrated that this soft physical twin allowed less invasive palpation while maintaining performance comparable to traditional methods. However, it was noted that surgeons required a learning period to achieve optimal accuracy. In a different approach, Doria et al. [32] developed a wearable tactile feedback device for myomectomy applications. The device utilised a fabric‐based system that transmitted stiffness information to the user's fingers by adjusting the indentation of the fabric through two DC motors placed above the finger. Extensive experiments with gynaecological surgeons in a silicon phantom model, designed to mimic uterine tissues with embedded leiomyomas, demonstrated the utility of this wearable system.

While soft displays offer more natural tactile sensations, they present distinct challenges in clinical implementation. The pneumatic actuation often requires complex infrastructure for air pressure control, which can be noisy and has limited resolution [34]. Users have reported that feedback from balloon‐based systems becomes more difficult to perceive over time, potentially impacting the consistency and reliability of the feedback [29]. Furthermore, these systems require precise control to simulate realistic tissue properties, and their effectiveness may be reduced when conveying high‐force interactions. Additionally, the durability and maintenance of soft displays can be a concern, as they may be prone to wear and tear, and require frequent replacement or calibration.

#### 3.2.3 Lateral Skin Stretch (SS)

**FIGURE 7:**
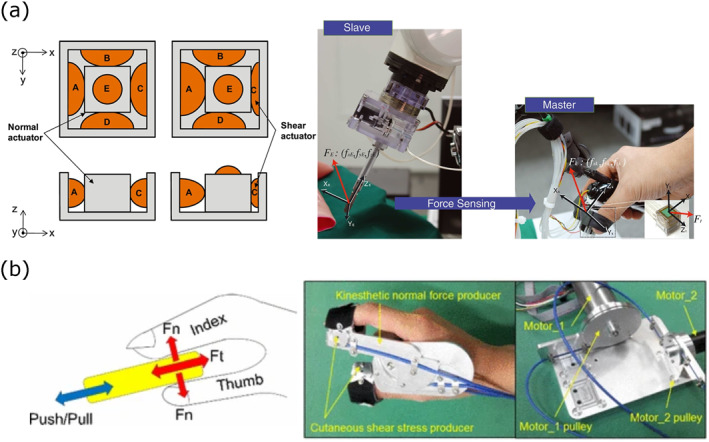
Lateral skin stretch devices. (a) Left: Stretch tactile display: *xy*‐directional movement via pressure difference between balloons A–C and B–D. Right: Force relationship between sensed and feedback forces. Adapted from Lim et al. [36] (ⓒ 2015 John Wiley & Sons Ltd. reprinted with permission). (b) Left: Forces generated during object grasping and manipulation. Right: Haptic device prototype. Adapted from Hu et al. 2016 [37] (ⓒ 2016 Springer Nature, reprinted with permission).

Lateral skin stretch has been explored to provide tactile feedback in RMIS, offering an additional dimension of sensory information that can enhance the surgeon's ability to perceive and manipulate tissues. This feedback modality allows for the simulation of forces such as friction and tension [35], which are critical for tasks such as needle insertion and tissue manipulation. A summary of SS devices is presented in Table 5, and Figure 7 illustrates various examples of skin stretching devices.

**TABLE 5:** Summary of studies utilising lateral skin stretch for skin deformation‐based tactile feedback in RMIS.

Reference	Tactile stimulus	Actuator	Additional stimulus	Location	Robotic interface	Robotic system	Application	Sensor	Virtual	Phantom	Ex vivo
Lim et al. [36]	NI‐S, SS	Pneumatic actuator	K, V	Finger	Phantom Premium 1.0 (3D Systems, USA)	Customised robot (RC7M, DENSO, Japan)	TM	Fiber Bragg grating (FBG), F/T sensor (Nano43, ATI, USA)	—	✓	—
Hu et al. [37]	NI‐R, SS	DC motor	V	Finger	In‐house hand interface	In‐house 2‐DoF forceps	NI	Strain gauges	—	✓	—
Han et al. [38]	SS	Electroactive polymer films	—	Finger	In‐house 1‐DoF linear interface	In‐house 1‐DoF needle insertion system	NI	Fiber Bragg grating (FBG)	—	✓	—
Quek, Provancher and Okamura [39]	NI‐R, SS	DC motor	K, V	Finger	da Vinci console interface	da Vinci Research Kit (Intuitive Surgical, USA)	TM	F/T sensor (Nano17, ATI, USA)	—	✓	—
Kossowsky, Farajian and Nisky [40]	SS	Not specified	K, V	Finger	Phantom Premium 1.5 (3D Systems, USA)	Virtual	PP	None	✓	—	—

Abbreviations: K, kinaesthetic; NI, needle insertion; NI‐R, normal indentation (rigid indenter); NI‐S, normal indentation (soft display); PP, palpation; SS, skin stretch; TM, telemanipulation; V, visual.

Lim et al. [36] developed a pneumatic 3‐axis tactile display capable of generating both two‐axis shear forces and normal forces on the thumb and index fingers (Figure 7a). This system was integrated with optical Fiber Bragg Grating (FBG) sensors mounted on surgical tool tips to measure tensile strain and thermal variations. The combination of kinaesthetic and tactile feedback provided by this system resulted in improved performance in teleoperated robot‐assisted surgical tasks. Further advancing the concept of skin stretch, Hu et al. [37] introduced a portable haptic device that delivers both kinaesthetic grasp force feedback and cutaneous push‐pull force feedback. Using a Bowden cable‐driven mechanism, this device maintained the free motion of the operator's hands while reproducing the sensed forces of the slave robot. In needle insertion tests, haptic feedback allowed users to stop earlier based on tactile cues rather than relying solely on visual information, significantly enhancing safety during the procedure. Focused on MR‐compatible systems, Han et al. [38] presented a haptic display that utilised electroactive polymer actuators to provide lateral skin stretch feedback at the fingertips during needle insertion. This device, designed for use between the thumb and index finger, conveyed tool‐tip forces measured by a biopsy needle equipped with Fiber Bragg Grating sensors. The system achieved high membrane detection success rates in teleoperated tasks with minimal impact on MR imaging quality. Quek et al. [39] explored a multi‐degree‐of‐freedom tactile feedback device that combined cutaneous normal force and tangential skin stretch to convey interaction force information during teleoperated surgical tasks. The proposed device is designed to be grasped with a precision grip using multiple fingerpads, providing detailed feedback on force direction and magnitude. The experimental results showed that this method improved task performance and situational awareness without negatively impacting concentration. In a related study, Kossowsky et al. [40] investigated the effects of artificial tactile noise and kinaesthetic noise on the perception of stiffness within a virtual reality environment. Their findings indicated that, while tactile noise did not significantly impact stiffness perception, kinaesthetic variability increased participants' uncertainty. However, the study also suggested that artificial skin stretch could improve perceived stiffness.

Lateral skin stretch devices, though effective for directional feedback, face several limitations in practical implementation. While these devices can convey tactile force information in multiple directions, they cannot fully reproduce natural tactile information such as contact area spread rate or pressure distribution [41]. The requirement for precise calibration and control to maintain effectiveness adds complexity to the system, and the higher interaction forces associated with skin stretch can contribute to user fatigue during extended procedures [38]. Additionally, lateral skin stretch devices typically require a larger number of actuators to control motion direction, amplitude, and frequency, which can increase the system's size, weight, and cost, making it more difficult to integrate into existing surgical systems [36].

### 3.3 Vibrotactile Feedback (VB)

**FIGURE 8:**
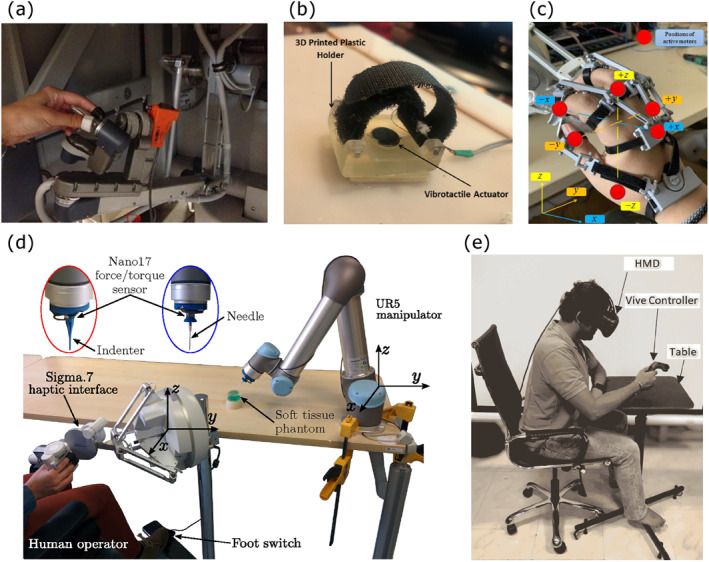
Vibrotactile feedback. (a) da Vinci master handle augmented with electromagnetic voice coil actuators for haptic display of instrument vibrations. Adapted from Koehn and Kuchenbecker [43] (ⓒ 2015 Springer Nature, reprinted with permission). (b) Shaftless vibrotactile actuator for remote palpation using a robotic surgical finger. Adapted from Hamdi et al. [53]. (c) Active motor positions in a wearable vibration glove. Adapted from Bai et al. [46]. (d) Master system with Sigma.7 haptic interface with vibrotactile feedback provided through oscillatory forces and slave system with force/torque sensor mounted on a UR5 robotic arm. Adapted from Meli, Pacchierotti and Prattichizzo [44] (ⓒ 2017 John Wiley & Sons Ltd. reprinted with permission). (e) VR‐based training system with Vive controller providing vibrotactile feedback via a linear resonant actuator for surgical training. Adapted from Vasudevan et al. [49] (ⓒ 2020 John Wiley & Sons Ltd. reprinted with permission).

Vibrotactile devices are commonly favoured for displaying tactile information in RMIS due to their low cost, versatility, and ability to provide effective sensory feedback. These devices use vibrations to convey important cues to the surgeon, enhancing the perception of tool‐tissue interactions. A summary of vibrotactile devices is presented in Table 6, and various examples of these devices can be found in Figure 8.

**TABLE 6:** Summary of studies utilising vibrotactile devices for tactile feedback in RMIS.

Reference	Tactile stimulus	Actuator	Additional stimulus	Location	Robotic interface	Robotic system	Application	Sensor	Virtual	Phantom	Ex vivo
Pacchierotti et al. [42]	VB	DC motor	K, V	Finger	Omega 6 (Force Dimension, Switzerland)	In‐house 2‐DOF needle steering	NI	Ultrasound transducer	—	✓	—
Koehn and Kuchenbecker [43]	VB	Voice coil	V, A	Finger	da Vinci console interface	da Vinci robot (Intuitive Surgical, USA)	TM	MEMS‐based accelerometer	—	✓	—
Meli, Pacchierotti, and Prattichizzo [44]	VB	DC motor	K	Hand	Sigma 7 (Force Dimension, Switzerland)	Customised robot (UR5, Universal Robot, Denmark)	PP, NI	F/T sensor (Nano17, ATI, USA)	—	✓	—
Pacchierotti et al. [45]	VB	DC motor	K, V	Finger	Omega 6 (Force Dimension, Switzerland)	Miniaturised soft gripper	TM	CCD sensor	—	✓	—
Bai et al. (2019) [46]	VB	Vibration motor	K	Hand	Falcon (Novint, USA)	Catheter robot	OA	None	—	✓	—
Abiri et al. [47]	VB	Vibration motor	V	Finger	da Vinci console interface	da Vinci robot (Intuitive Surgical, USA)	OA	Force sensor (FlexiForce A101, Tekscan, USA)	—	✓	—
D'Abbraccio et al. [48]	VB	Piezoelectric actuator	V	Finger	In‐house hand interface	Certesian manipulator (STANDA)	PP	F/T sensor (Nano43, ATI, USA)	—	✓	—
Vasudevan et al. [49]	VB	—	V	Hand	VR controller (HTC Vive)	Virtual	OA	None	✓	—	—
Mendelsohn et al. [50]	VB	Vibration motor	V	Finger	da Vinci console interface	da Vinci robot (Intuitive Surgical, USA)	OA	Force sensor (FlexiForce A101, Tekscan, USA)	—	✓	—
Aggravi et al. [51]	VB	Vibration motor	K, V	Forearm	Omega 6 (Force Dimension, Switzerland)	Customised robot (Viper S650 Adept, Omron, Japan)	NI	F/T sensor (Nano43, ATI, USA), Ultrasound scanner (SonixTOUCH, Ultrasonix, UK)	—	✓	—
Tahir et al. [52]	VB	Vibration motor	K, V	Hand	In‐house 2‐DoF interface	In‐house 2‐DoF guidewire device	OA	None	—	✓	—
Hamdi et al. [53]	VB	Vibration motor	V	Finger	In‐house hand interface	In‐house 3‐DoF finger	PP	Force sensor (FSR, Tekscan, USA)	—	✓	—

Abbreviations: A, auditory; K, kinaesthetic; NI, needle insertion; OA, other application; PP, palpation; TM, telemanipulation; V, visual; VB, vibrotactile.

One of the early studies, Pacchierotti et al. [42] introduced a teleoperated robotic system to steer flexible needles that combined kinaesthetic and vibratory feedback. The system used an ultrasound imaging system to track needle position and compute the ideal trajectory. Surgeons received navigation cues through the master haptic interface, which provided both kinaesthetic and vibratory forces. Experimental validation with 20 participants revealed that vibratory feedback was more effective than visual feedback in conveying navigation cues, achieving a mean targeting error of just 0.72 mm. Koehn and Kuchenbecker [43] explored the value of vibration feedback in robotic surgery by conducting human‐subject studies with both surgeons and non‐surgeons (Figure 8a). Participants used an augmented da Vinci robot to perform dry‐lab tasks with and without haptic and audio feedback. Results from both experiments, involving 10 surgeons and 10 non‐surgeons showed an overwhelming preference for receiving vibration feedback. Participants reported that the feedback increased their awareness of tool contacts without interfering with their use of the robot. In another study, Meli, Pacchierotti, and Prattichizzo [44] evaluated a teleoperation system that provided magnified haptic feedback through a 7‐DoF haptic interface (Figure 8d). This system, which also controlled a 6‐DoF slave robotic manipulator, significantly enhanced the clinician's ability to discriminate stiffness, with performance improvements of up to 306% during needle insertion and 80% during palpation, compared to direct hand interaction. Pacchierotti et al. [45] later introduced a microteleoperation system designed for the intuitive steering of miniaturised self‐folding soft magnetic grippers. The system provided haptic sensations, including vibrotactile feedback, to the operator based on the interaction between the gripper and the environment. The study demonstrated that haptic feedback, including vibrotactile cues, significantly improved performance in navigation and micromanipulation tasks. Bai et al. [46] developed a sensor‐less method for estimating contact torque and providing vibrotactile feedback in catheter robots used for minimally invasive surgery. By estimating contact torque and motion of the motors using a quasi‐statics model and delivering feedback through a wearable vibrotactile device (Figure 8c), the system guided surgeons to effectively avoid tissue contact, with maximum errors under 5% across different phases.

Vibrotactile feedback has also been utilised to enhance specific surgical tasks. Abiri et al. [47] introduced a vibrotactile feedback system aimed at reducing suture breakage and improving knot quality during robotic‐assisted knot tying. The system provided feedback to the operator's fingertips when suture tension approached failure thresholds, significantly reducing instances of suture failure and enhancing knot quality. D'Abbraccio et al. [48] developed a tele‐palpation apparatus enabling users to detect nodules with varying stiffness within a polymeric phantom. The system used a haptic glove embedded with a piezoelectric disk to provide vibrotactile feedback, which was encoded using a neuromorphic model. The study showed a high identification accuracy for stiffer inclusions. Vasudevan et al. [49] highlighted the importance of vibrotactile feedback in a virtual reality‐based training system for robotic surgery (Figure 8e). Their study found that vibrotactile feedback improved the performance of manipulation tasks by reducing reaction times, particularly when the visual system was overloaded, underscoring the effectiveness of tactile cues over visual feedback in training environments. Similarly, Mendelsohn et al. [50] incorporated vibrotactile feedback into a transoral robotic surgery (TORS) training platform to provide real‐time tactile collision awareness. The feedback system, which activated vibration motors after detecting major collisions, significantly reduced the number of major anatomic collisions and improved surgical proficiency, as measured by higher Global Evaluative Assessment of Robotic Skills (GEARS) scores and better tumour resection outcomes. Aggravi et al. [51] proposed a haptic‐enabled teleoperation system for flexible needle insertion in soft tissue. The system combined 3D ultrasound guidance with kinaesthetic and vibrotactile feedback, delivering navigation cues through a grounded kinaesthetic device and needle tip cutting force feedback through a wearable vibrotactile armband. The study demonstrated a 87% improvement in accuracy when both feedback modalities were used together, compared to the absence of haptic feedback. Tahir et al. [52] explored a novel haptic guidance procedure for tele‐operated coronary interventions. Their system combined kinaesthetic and vibrotactile feedback, utilising x‐ray images for guidewire navigation. The study found that vibrotactile feedback significantly reduced task completion time, especially in navigating challenging coronary branches, making it more effective than kinaesthetic feedback alone. Hamdi et al. [53] developed a 3D printed robotic surgical finger with an integrated vibrotactile feedback system controlled by a master glove (Figure 8b). This system, using a shaftless eccentric mass motor, mimicked the movements of the surgeon's fingers to distinguish between different materials that mimic human flesh, tumour and bone, enhancing the surgeon's ability to perform finger dissection in both laparoscopic and robotic surgery.

Despite its widespread use, vibrotactile feedback presents significant limitations in surgical applications. Koehn and Kuchenbecker [43] observed that prolonged exposure to vibration can lead to decreased tactile sensitivity and increased user fatigue. Additionally, single vibrotactile actuators can only display force magnitude and frequency information, resulting in the loss of important directional signals [41]. Furthermore, the limited dynamic range of vibrotactile actuators can also limit the range of tactile sensations that can be conveyed.

### 3.4 Multi‐Modal Tactile Feedback

Multi‐modal tactile feedback systems, which combine various types of tactile stimulus modalities such as skin deformation and vibrotactile stimuli, have been explored as a means to enhance the sensory experience in RMIS. These systems aim to provide a more comprehensive sense of touch, improving the surgeon's ability to perceive and manipulate tissues accurately. Table 7 provides a summary of related works on multi‐modal tactile feedback.

**TABLE 7:** Summary of studies utilising multimodal tactile feedback in RMIS.

Reference	Tactile stimulus	Actuator	Additional stimulus	Location	Robotic interface	Robotic system	Application	Sensor	Virtual	Phantom	Ex vivo
Pacchierotti et al. [54]	NI‐R, VB	Servomotor, vibration motor	V	Finger	da Vinci console interface	da Vinci robot (Intuitive Surgical, USA)	PP	BioTac tactile sensor (SynTouch, USA)	—	✓	—
Abiri et al. [55]	NI‐S, VB	Pneumatic actuator, vibration motor	V	Finger	da Vinci console interface	da Vinci robot (Intuitive Surgical, USA)	PP	Force sensor (FlexiForce A201, Tekscan, USA)	—	✓	—
Abiri et al. [56]	NI‐S, VB	Pneumatic actuator, vibration motor	V	Finger	da Vinci console interface	da Vinci robot (Intuitive Surgical, USA)	TM	Force sensor (FlexiForce A201, Tekscan, USA)	—	✓	✓
Ouyang et al. [57]	NI‐S, VB	Pneumatic actuator, vibration motor	V	Finger	da Vinci console interface	da Vinci robot (Intuitive Surgical, USA)	PP	Force sensor (FlexiForce A201, Tekscan, USA)	—	✓	—

Abbreviations: NI‐R, normal indentation (rigid indenter); NI‐S, normal indentation (soft display); VB, vibrotactile; V, visual; TM, telemanipulation; PP, palpation.

**FIGURE 9:**
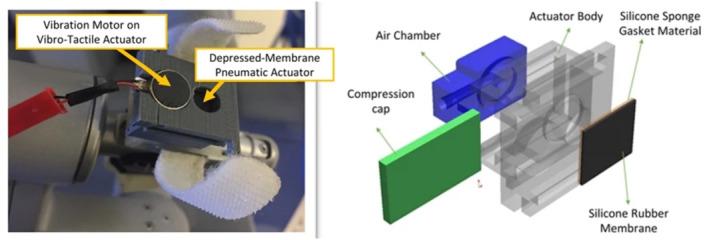
Multimodal tactile feedback components. Left: Vibration motor and pneumatic actuator integrated into da Vinci controller. Right: CAD model of a 3D‐printed depressed membrane tactile actuator. Adapted from Abiri et al. [56].

Pacchierotti et al. [54] introduced an innovative cutaneous feedback system for the da Vinci surgical robot, designed to provide surgeons with real‐time feedback on fingertip contact deformation and vibrations. The system integrates a BioTac tactile sensor mounted on one of the robot's slave tools with a custom cutaneous display attached to the corresponding master controller. Contact deformations and vibrations detected by the BioTac sensor are mapped directly to the cutaneous device's motors using a model‐free algorithm based on look‐up tables. This approach was tested in a palpation task, where participants were asked to detect the orientation of a plastic stick embedded in a simulated heart tissue model. The study showed that providing cutaneous feedback significantly improved task performance, reducing the absolute error in detecting the stick's orientation, as well as improving completion time and reducing the pressure exerted on the model. Expanding on the concept of multi‐modal feedback, Abiri et al. [55] evaluated a bimodal vibrotactile system that conveyed applied forces to simulate haptic feedback in an artificial palpation task using the da Vinci surgical robot. Their study included two experimental setups: the first involved localising an embedded vessel in a soft tissue phantom using a single‐sensor unit, and the second involved localising tumour‐like structures using a three‐sensor array. Both setups were tested under three conditions: no feedback, normal force tactile feedback, and hybrid vibrotactile feedback. The addition of vibrotactile and pneumatic feedback significantly improved the accuracy of correct localization attempts. In a following study Abiri et al. [56] developed a comprehensive multi‐modal haptic feedback system that integrates tactile, kinaesthetic, and vibrotactile feedback to augment the sense of touch in robotic surgery (Figure 9). This system was evaluated using the da Vinci surgical robot in both peg transfer tasks and ex vivo bowel tissue handling experiments. The results demonstrated that the multi‐modal feedback system significantly reduced both average and peak grip forces applied by users, bringing them closer to the levels typically achievable with the human hand. This reduction in force application was observed in both novice and expert users, indicating that the system could help surgeons achieve more delicate and precise manipulation during surgery. Ouyang et al. [57] proposed a bio‐inspired control model that combined pneumatic balloons and vibration motors to provide force feedback and vibration, helping surgeons localise underlying structures in phantom tissue. The system used outputs from a model of cutaneous afferents based on pressure signals from a sensor embedded in surgical forceps to control the actuators. In a study involving 25 participants, including 10 expert surgeons, the bio‐inspired feedback method proved more sensitive than traditional control methods, especially in recognising soft tumours. This system allowed both novices and experts to more easily identify the locations of various tumour types, with reduced contact force and tumour contact time compared to previous control functions or when no haptic feedback was provided.

While combining different feedback modalities can enhance performance, multimodal systems face unique challenges. The increased complexity of these systems can lead to higher costs, maintenance requirements, and potential conflicts between different feedback types [33]. Furthermore, the simultaneous activation of multiple feedback modalities may not correspond to natural tactile sensations, potentially impacting user experience and system effectiveness [57].

### 3.5 Comparison Between Tactile Feedback Modalities

Other studies have explored the relative strengths and weaknesses of various feedback methods, providing valuable insights into how these modalities can be best utilised in surgical applications. A summary of these studies is presented in Table 8.

**TABLE 8:** Summary of studies comparing tactile feedback modalities in RMIS.

Reference	Tactile stimulus	Actuator	Additional stimulus	Location	Robotic interface	Robotic system	Application	Sensor	Virtual	Phantom	Ex vivo
Schorr et al. [41]	SS, VB	DC motor	K, V	Finger	Phantom Premium 1.5 (3D Systems, USA)	Phantom Premium 1.5 (3D Systems, USA)	PP	F/T sensor (Nano17, ATI, USA)	—	✓	—
Ferro et al. [58]	NI‐R, SS, VB	Vibration motor, Piezoelectric actuator	K, V	Finger, Forearm	Geomagic Touch (3D Systems, USA)	Virtual	NI	None	✓	—	—

Abbreviations: K, kinaesthetic; NI, needle insertion; NI‐R, normal Indentation (rigid indenter); PP, palpation; SS, skin stretch; V, visual; VB, vibrotactile.

Schorr et al. [41] conducted a study to evaluate the efficacy of tactor‐induced skin stretch as a sensory substitution method in teleoperated palpation tasks. The study compared skin stretch feedback with traditional force feedback, reduced gain force feedback, graphical feedback, and vibration feedback. The results demonstrated that skin stretch feedback was as effective as force feedback in determining the orientation of stiff regions within artificial tissue models. However, it was observed that skin stretch feedback led to higher interaction forces. Despite this, the study highlighted that skin stretch feedback could effectively convey both force magnitude and direction information, similar to kinaesthetic force feedback, with the added advantage of preserving system stability, which is often a concern with force feedback systems.

In a more recent study, Ferro et al. [58] focused on the effectiveness of different types of haptic feedback during needle insertion tasks in soft tissues, conducted through a remotely operated robot. The study involved three experiments with human subjects to analyse the impact of grounded kinaesthetic feedback, cutaneous vibrotactile feedback, and cutaneous pressure feedback on rendering the elastic and viscous force components of a needle‐tissue interaction model. The results indicated that the best performance was achieved when the two types of feedback information, elastic and viscous, were provided through different channels, such as kinaesthetic and cutaneous feedback. This approach was more effective than delivering both types of information through a single commercial grounded kinaesthetic interface. Furthermore, their study revealed that cutaneous pressure feedback was particularly well‐suited for rendering the elastic component of the interaction, outperforming the vibrotactile feedback. Interestingly, the study also suggested that the precise location of where the elastic feedback is rendered, whether at the user's interface grip or elsewhere, was not critical. This finding implies that delocalised cutaneous sensations can be an effective and flexible solution in haptic feedback systems, allowing greater versatility in system design.

## 4 Discussion

Despite substantial research efforts aimed at developing reliable tactile feedback systems for RMIS, most of these technologies, including skin deformation devices and vibrotactile feedback systems, have yet to be widely adopted in clinical practice due to challenges in integration, technical reliability, and cost‐effectiveness [21]. In this section, we will discuss the applications of these technologies, the challenges identified in previous studies, and the factors that continue to hinder their clinical implementation.

### 4.1 Applications in RMIS

Experimental studies have shown that the integration of tactile feedback in robot‐assisted minimally invasive surgery (RMIS) significantly enhances performance across various surgical tasks, including palpation, telemanipulation, needle insertion, and surgical training. The addition of tactile feedback provides surgeons with crucial sensory information that can improve accuracy and overall efficiency during procedures.

#### 4.1.1 Palpation

Palpation is a crucial aspect of RMIS, especially for identifying tumours, whose increased rigidity often distinguishes them from healthy tissues. Accurate tactile feedback is essential for discerning these differences and ensuring precise tumour localization. Many studies in this review have focused on enhancing palpation by providing tactile feedback that mimics the sensation of tissue stiffness. For instance, rigid tactors have been developed to simulate stiffness as a normal force applied to the surgeon's fingertips, effectively replicating the tactile experience of probing tissues with varying densities [27].

In addition, soft surfaces using pneumatic actuators have been explored to convey information about the characteristics of softer tissue. These advancements include the development of digital twins that accurately model tissue properties [33], as well as multi‐finger feedback systems that offer more nuanced control and sensory feedback [31]. Vibrotactile feedback has also been embedded in wearable interfaces, allowing surgeons to receive real‐time tactile information about tissue stiffness while simultaneously controlling robotic systems [48, 53].

Moreover, multimodal tactile feedback systems that combine normal indentation with vibrotactile feedback have been investigated for palpation in RMIS. These systems provide a richer and more comprehensive sensory experience, leading to improvements in task completion times and a reduction in false detections compared to single‐modality tactile feedback and purely kinaesthetic feedback [54, 55, 57].

#### 4.1.2 Telemanipulation

In telemanipulation, the integration of various haptic stimuli has been shown to be highly effective in enhancing the surgeon's ability to perform precise and safe surgical manoeuvres [22]. Telemanipulation of tissues and organs relies heavily on accurately estimating their dynamic characteristics, including stiffness and elasticity [59]. Surgeons often depend on grip force feedback to make these estimations, which is critical to avoiding tissue damage and ensuring successful outcomes.

Several studies have explored the use of normal indentation with rigid (NI‐R) tactors within wearable devices [25] and soft (NI‐S) displays embedded into the da Vinci console interface [9, 29, 30]. These approaches have shown a substantial decrease in grip forces, thereby minimising tissue damage during manipulation. Additionally, skin stretch devices have been investigated as a means to provide directional information that complements kinaesthetic feedback, improving the accuracy of pulling force control. Studies have shown that tactile information from skin stretch devices is more effective at recognising small forces compared to kinaesthetic feedback alone [36].

Research has also demonstrated that using a 3‐DOF skin deformation (SD) tactile device in combination with visual and kinaesthetic feedback can achieve lower or comparable forces and shorter trial times in telemanipulation tasks, such as peg transfer, needle passing, and circle cutting, compared to using only visual or kinaesthetic feedback [39].

Vibrotactile feedback has been successfully integrated into the da Vinci console interface to replicate instrument vibrations on the user's fingertips during telemanipulation tasks. This feedback modality has been particularly favoured by surgeons, especially for tasks like needle passing, where it was preferred over auditory feedback [56]. Furthermore, multimodal tactile feedback systems that combine pneumatic and vibrotactile stimuli with kinaesthetic feedback have shown significant reductions in both average and peak forces during peg transfer tasks using the da Vinci robot, highlighting the benefits of a hybrid approach in enhancing telemanipulation performance [56].

#### 4.1.3 Needle Insertion

In needle insertion tasks within RMIS, the combination of tactile and kinaesthetic feedback has been shown to significantly improve performance, particularly in terms of reducing penetration forces and avoiding undesired tissue regions. For instance, a 3‐DOF normal indentation with rigid (NI‐R) device was proposed by Pacchierotti et al. [26], demonstrating that the integration of tactile feedback with kinaesthetic cues can lead to more controlled and precise needle insertions in soft tissues.

Skin stretch devices have also been explored for needle insertion tasks, leveraging the directional information they provide to enhance the surgeon's ability to control push/pull forces. Studies by Hu et al. [37] and Han et al. [38] have shown that these devices can effectively render the elastic and friction forces encountered during needle insertion into tissue phantoms with embedded membranes. This additional feedback helps surgeons maintain control over the needle trajectory, reducing the likelihood of unintended tissue damage.

Vibrotactile feedback has also been integrated into kinaesthetic devices to provide oscillatory force feedback, which helps constrain needle motion and avoid specific tissue regions. Meli, Pacchierotti and Prattichizzo [44] demonstrated the utility of this approach in guiding needle insertion, ensuring that the needle remains within safe boundaries. Furthermore, Aggravi et al. [51] introduced vibrotactile feedback delivered to the forearm to simulate cutting and friction forces during flexible needle insertion into a gelatin phantom. Their results indicated that the combination of kinaesthetic and vibrotactile feedback consistently outperformed the use of either modality alone, reinforcing the value of multimodal feedback in enhancing surgical precision, a finding echoed by other studies [42].

Additionally, a recent study by Ferro et al. [58] compared the effectiveness of various tactile feedback modalities in a simulated needle insertion task. Their findings revealed that pressure‐based cutaneous sensations were particularly effective in conveying the elastic properties of the interaction, while vibrotactile feedback was less suitable for transmitting this type of information, suggesting that different types of feedback may be better suited to specific aspects of needle insertion.

#### 4.1.4 Other Applications

Tactile feedback has also been explored in various other critical applications within RMIS, such as suturing and surgical training. In suturing tasks, precise control of tension forces is essential to prevent damage to delicate tissues. Abiri et al. [47] developed a vibrotactile feedback system that uses vibration motors to provide real‐time warnings to surgeons about the force applied to the sutures. This system, integrated into the da Vinci robotic platform, alerts the surgeon through tactile feedback at the fingertips, helping to maintain appropriate force levels during suturing and knot tying, thereby reducing the risk of tissue damage.

Surgical training has also benefited from the integration of tactile feedback, particularly in enhancing fine motor skills and collision awareness. Vasudevan et al. [49] investigated the use of vibrotactile feedback in virtual reality (VR) interfaces for training surgeons in fine motor skills. This approach provided learners with a more immersive and realistic training environment, improving their ability to perform precise tasks in a virtual environment. Similarly, Mendelsohn et al. [50] explored the use of real‐time tactile feedback to enhance collision awareness during transoral robotic surgical training. By providing tactile cues to indicate proximity to critical structures, this feedback mechanism helps trainees avoid unintended collisions, thereby improving their surgical precision and safety.

### 4.2 Limitations of Tactile Feedback Modalities

Although tactile feedback technologies show promise in robot‐assisted minimally invasive surgery (RMIS), they face several limitations, particularly regarding usability, ergonomic integration, and comparative effectiveness with kinaesthetic feedback. These limitations impact long‐term utility and overall user experience, as summarised in Table 9 and detailed below.

**TABLE 9:** Disadvantages of tactile feedback modalities.

Tactile feedback modality	Disadvantages
Skin deformation (rigid)	– May cause discomfort, particularly during prolonged use or when applying high forces – Can result in a reduced contact area and decreased sensitivity. – Mechanical properties of rigid displays can introduce unwanted vibrations, noise, and mechanical artefacts
Skin deformation (soft)	– Requires precise control to simulate realistic tissue properties and maintain effectiveness – May be less effective in conveying high‐force interactions and have limited durability – Integration with existing systems can be complex and require additional infrastructure
Skin deformation (stretch)	– Limited ability to convey contact area spread and pressure distribution – Requires precise calibration and control to maintain effectiveness and may require additional actuators – Higher interaction forces associated with skin stretch can contribute to user fatigue during extended procedures.
Vibrotactile feedback	– Can cause decreased tactile sensitivity and muscle fatigue over extended use – May become distracting or less effective over time due to adaptation of mechanoreceptors – Limited ability to convey nuanced force information, spatial resolution, and directional signals
Multimodal feedback	– Increased system complexity, cost, and maintenance requirements – Potential for conflicting sensory inputs, sensory overload, and decreased user performance – Requires careful consideration of relative importance and priority of each modality

#### 4.2.1 Usability and Ergonomic Considerations

The implementation of tactile feedback in RMIS presents significant usability and ergonomic challenges that can impact surgical performance and user comfort. These challenges primarily manifest in three primary areas: physical fatigue and comfort, device integration constraints, and user adaptation requirements.

##### 4.2.1.1 Physical Fatigue and Comfort

Extended use of tactile feedback systems can lead to user fatigue and decreased effectiveness. Vibrotactile feedback, while initially effective, has been shown to cause discomfort and reduced tactile sensitivity during prolonged use [60]. Similarly, Aggravi et al. [51] reported that repeated vibrotactile signals can become unpleasant and potentially degrade performance over time. Similarly, skin stretch feedback, though effective for directional information, has been associated with higher interaction forces that may contribute to muscle fatigue [41].

##### 4.2.1.2 Integration and Design Constraints

Physical integration of tactile displays into surgical interfaces presents significant ergonomic challenges. Devices must balance functionality with size and weight constraints to ensure unobtrusive interaction [32]. Multiple actuators often result in bulky designs [54], while wearable tactile devices can impede natural hand movements during lengthy procedures [25]. Hu et al. [37] emphasise the importance of lightweight designs to reduce inertia and improve portability, particularly in multi‐modal systems where additional hardware can compromise ergonomic efficiency [56].

##### 4.2.1.3 Individual Variability and Adaptation

User response to tactile feedback varies significantly among individuals, necessitating adaptable systems. Schorr et al. [41] observed considerable inter‐participant variability in perceiving skin stretch cues, attributed to differences in skin stiffness and tactile sensitivity. To address these individual differences, Koehn and Kuchenbecker [43] suggest implementing adjustable feedback settings that can be customised based on user preferences and procedure requirements. The effectiveness of feedback may also vary depending on body location and rendering parameters [58], highlighting the need for careful consideration of these factors in system design.

##### 4.2.1.4 Cognitive Load and Usability

While tactile feedback can help in reducing excessive grip forces, particularly among non‐expert surgeons, the existing visual–perceptual mismatch can still impose a cognitive load. Abiri et al. [30] demonstrated that despite tactile feedback assistance, surgeons may experience increased cognitive demands in maintaining appropriate grip forces throughout a task, especially when visual cues are insufficient or misleading.

#### 4.2.2 Comparative Effectiveness With Kinaesthetic Feedback

The effectiveness of tactile versus kinaesthetic feedback in RMIS presents complex trade‐offs between performance benefits and implementation challenges. While kinaesthetic feedback provides intuitive force guidance, it faces significant technical limitations that tactile feedback aims to address. A primary challenge with kinaesthetic feedback is the inherent trade‐off between system stability and transparency. Force feedback in master‐slave systems can create uncontrollable oscillations due to closed‐loop coupling between user input and feedback forces [34]. Additionally, high friction and inertia in kinaesthetic systems can mask small forces and cause user fatigue during fine tasks [25]. In contrast, tactile feedback systems offer simpler integration and enhanced stability, though they may provide less comprehensive feedback.

This difference in implementation complexity directly affects performance across various force ranges and tasks. Studies comparing tactile and kinaesthetic feedback have revealed task‐dependent effectiveness. Lim et al. [36] demonstrated that tactile feedback outperforms at conveying small forces, while kinaesthetic feedback proves more effective for larger forces. This distinction becomes particularly relevant in surgical tasks requiring precise force control. While tactile feedback may provide less realistic sensation than kinaesthetic feedback [26], it has shown superior performance in specific applications like softness discrimination [32].

The task‐specific nature of feedback effectiveness becomes even more apparent across different surgical procedures. In needle insertion tasks, for instance, kinaesthetic feedback provides better guidance while tactile feedback more effectively conveys cutting forces [51]. For structure localization, combining vibrotactile with kinaesthetic feedback significantly improves performance compared to either modality alone [55]. This complementary relationship extends to training applications, where tactile feedback serves as an effective trigger for developing kinaesthetic memory, particularly beneficial for novice users [29].

Given the distinct strengths and limitations of kinaesthetic and tactile feedback, evidence increasingly suggests that combining these modalities can yield superior results in surgical applications, especially in tasks that require precise force control [58]. Multi‐modal systems have shown improved performance in force regulation [56], though careful integration is necessary to avoid overwhelming users with complex sensory information. Moving forward, the primary challenge lies in determining the optimal combination of feedback types for specific surgical tasks, balancing enhanced performance with system simplicity, stability, and user comfort.

At the same time, a fundamental question remains regarding the extent of tactile feedback necessary for effective surgical assistance. While recreating a full range of tactile sensations may seem advantageous, evidence suggests that targeted feedback, focusing on key tactile cues, could achieve comparable performance gains with reduced system complexity. For instance, Koehn and Kuchenbecker [43] noted that experienced surgeons, often accustomed to operating without haptic feedback, may show a preference for familiar techniques, indicating that extensive sensory augmentation may not always be required. These insights emphasise the need to balance technological capabilities with practical requirements, aiming to identify the tactile feedback elements that offer the greatest clinical benefit rather than pursuing comprehensive sensory recreation.

### 4.3 Design Challenges and Considerations

The development of tactile displays for robot‐assisted minimally invasive surgery (RMIS) presents unique and complex challenges. Unlike traditional laparoscopic surgery, where surgeons have direct contact with surgical instruments, RMIS involves the use of robotic systems controlled remotely via interfaces. This separation between the surgeon and the surgical site amplifies the difficulty in providing effective tactile feedback. For tactile displays to be effectively integrated into robotic interfaces, they must overcome challenges similar to those faced in tactile sensing, as well as several unique obstacles.

#### 4.3.1 Complexity and Variety of Tactile Sensations

Tactile feedback encompasses a broad range of sensations, including pressure, texture, and vibration, each requiring different types of stimuli to be accurately rendered. The need to reproduce these sensations naturally and seamlessly without overloading the surgeon's cognitive capacity adds a layer of complexity to the development of tactile displays.

#### 4.3.2 Passive Integration

Passive integration of tactile feedback in RMIS aims to provide additional sensory information without interfering with the primary kinaesthetic feedback from the robotic system. This independence ensures that tactile feedback enhances the surgeon's perception of tissue properties and interactions without disrupting the overall control of the robotic system.

#### 4.3.3 Transparency

Tactile displays must provide information in a natural and intuitive manner, ensuring that the surgeon can perceive tactile cues without increasing cognitive workload. Achieving this transparency is challenging, as the feedback must be both accurate and timely, integrating smoothly with the visual and kinaesthetic inputs that the surgeon relies on.

#### 4.3.4 Compactness and Weight

Tactile displays used in RMIS must be compact enough to be integrated into the robotic interface without impeding surgeon's control of the robotic arms. These systems should be lightweight to avoid adding unnecessary inertia or resistance to interface controls, which could reduce surgeon precision and increase fatigue. The design of these systems must account for space constraints within the control consoles and ensure that they do not interfere with the surgeon's ability to operate the robotic system effectively.

#### 4.3.5 Time Delay

The delay between the detection of tactile sensations by robotic instruments and the delivery of feedback to the surgeon must be minimised. In RMIS, even slight delays can disrupt the surgeon's ability to perform delicate tasks, as lack of real‐time feedback can lead to errors in judgement and execution. Therefore, reducing latency in tactile feedback systems is critical to maintaining the accuracy and effectiveness of robotic‐assisted surgery.

#### 4.3.6 Safety

The tactile feedback provided through RMIS interfaces must be carefully controlled to ensure that the forces generated are safe for both the surgeon and the patient. Excessive feedback forces could cause the surgeon to inadvertently apply too much pressure, potentially harming delicate tissues. Thus, the tactile display must be designed to provide precise, safe feedback that enhances surgeon control without posing additional risks.

#### 4.3.7 Adjustability

Different surgeons may require different levels of tactile stimulation based on their preferences and sensitivities. Therefore, tactile displays should offer adjustable feedback settings to cater to individual needs, enhancing comfort and effectiveness.

#### 4.3.8 Sterilisation

The components of the tactile display that are integrated into the RMIS console or any elements that come into contact with the surgical environment must be sterilisable. Ensuring that these components can be easily sterilised without degrading their performance or lifespan is essential.

#### 4.3.9 Reusability and Cost

In RMIS, tactile display systems should be designed for durability and reusability to be cost‐effective. While single‐use devices might simplify sterilisation, they increase operational costs and environmental impact. Therefore, developing reusable tactile displays that maintain their performance over multiple uses is important for both economic and practical reasons.

### 4.4 Clinical Validation and Pathways for Adoption

The translation of tactile feedback technologies from research settings to clinical practice is a complex process that extends beyond technical development. Despite these challenges, recent advancements have led to the integration of haptic systems into commercial surgical systems. For instance, the Saroa surgical system [61] has achieved clinical implementation of haptic systems, providing real‐time haptic force feedback to surgeons. This system allows surgeons to feel an object's hardness when grasped with forceps, and the feedback function can be adjusted or turned off as needed. Similarly, Intuitive Surgical has announced plans to incorporate haptic feedback into the Da Vinci 5 system in 2025. These cases provide useful reference points for facilitating the clinical adoption of additional tactile feedback modalities. To facilitate widespread clinical adoption, the following key aspects must be considered:

- Regulatory and Safety Compliance: Systems must meet established safety and reliability standards and comply with regulatory requirements regarding biocompatibility of materials used in tactile interfaces.
- Cost‐Effectiveness: Evaluating implementation costs against proven clinical benefits is important for healthcare institutions.
- Training and Education: Structured training programs are necessary to familiarise surgical teams with the specific functionalities of tactile feedback systems.
- Workflow Integration: Effective integration with current surgical workflows is necessary to prevent disruption of established procedures.
- Scalability and Accessibility: Systems should be designed for scalability and accessibility across a variety of healthcare settings to maximise impact.

## 5 Conclusions

In robot‐assisted minimally invasive surgery (RMIS), surgeons' physical separation from the patient demands the development of advanced tactile display technologies to recreate lost sensations. This systematic review aimed to identify and evaluate tactile display technologies proposed and experimentally validated for restoring tactile sensations in RMIS. Our analysis highlights various tactile feedback modalities explored, including skin deformation, vibrotactile, and multimodal approaches, and discusses their applications in key surgical tasks such as palpation, telemanipulation, and needle insertion.

However, despite the potential of tactile feedback to enhance surgical performance in RMIS, the number of studies in this area has not shown a significant upward trend, indicating that this remains an open and challenging problem. To overcome these challenges, it is essential to address the limitations of current tactile feedback systems, including the need for high‐sensitivity, precise sensing, and compact, safe, responsive, and sterilisable systems. Furthermore, effective integration of tactile feedback into RMIS platforms and clinical environments requires careful consideration of regulatory compliance, cost‐effectiveness, seamless workflow integration, and comprehensive training programs. Rigorous clinical validation is also crucial to establish the reliability and safety of these technologies.

It is essential to acknowledge the limitations of this review. The exclusion of studies outside of the selected databases and the focus on experimental validations in ex vivo or phantom environments may limit the generalisability of the findings. Future reviews could expand the scope to include emerging technologies and broader search criteria, providing a more comprehensive understanding of the evolving landscape of tactile feedback in RMIS.

In conclusion, by addressing these challenges and limitations, future advancements can effectively enhance the precision and safety of robot‐assisted surgeries, providing surgeons with a more immersive sensory experience and ultimately leading to improved patient outcomes.

## Author Contributions

Conception and design: J. Colan; Acquisition and interpretation of the data: J. Colan, A. Davila; Writing and editing: J. Colan, A. Davila; Project Supervision and Funding Acquisition: Y. Hasegawa.

## Ethics Statement

The authors have nothing to report.

## Consent

The authors have nothing to report.

## Conflicts of Interest

The authors declare no conflicts of interest.

## Data Availability

Data sharing is not applicable to this article as no new data were created or analysed in this study.
